# Prognostic Role of Invasion-Related Extracellular Matrix Molecules in Diffusely Infiltrating Grade 2 and 3 Astrocytomas

**DOI:** 10.3390/brainsci14111157

**Published:** 2024-11-20

**Authors:** László Szivos, József Virga, Zoltán Mészár, Melinda Rostás, Andrea Bakó, Gábor Zahuczki, Tibor Hortobágyi, Álmos Klekner

**Affiliations:** 1 Department of Neurosurgery, University of Debrecen, H-4032 Debrecen, Hungary or szivos.laszlo@med.u-szeged.hu (L.S.); virga.jozsef@med.unideb.hu (J.V.); 2 Department of Neurosurgery, University of Szeged, H-6725 Szeged, Hungary; 3 Department of Oncology, University of Debrecen, H-4032 Debrecen, Hungary; dr.bako.andrea@med.unideb.hu; 4 Department of Anatomy, Histology and Embryology, University of Debrecen, H-4032 Debrecen, Hungary; meszarz@anat.med.unideb.hu; 5 Department of Biochemistry and Molecular Biology, University of Debrecen, H-4032 Debrecen, Hungary; rostas.melinda@med.unideb.hu; 6 UD-GenoMed Medical Genomic Technologies Ltd., H-4032 Debrecen, Hungary; zahu@med.unideb.hu; 7 Department of Neurology, University of Debrecen, H-4032 Debrecen, Hungary; hortobagyi@med.unideb.hu; 8 Institute of Neuropathology, University Hospital Zurich, 8091 Zurich, Switzerland

**Keywords:** peritumoral infiltration, astrocytoma, invasion spectrum, extracellular matrix, prognostic factor

## Abstract

Background: Astrocytoma, an IDH-mutant is a common primary brain tumor. Total surgical resection is not feasible due to peritumoral infiltration mediated by extracellular matrix (ECM) molecules. Methods: This study aimed at determining the expression pattern of ECM molecules in different prognostic groups of WHO grade 2 and grade 3 patients and identifying the effect of onco-radiotherapy on tumor cell invasion of grade 3 patients. Gene and protein expression of ECM molecules was determined by qRT-PCR and immunohistochemistry, respectively. Results: In the different prognostic groups of grade 2 tumors HMMR, IDH-1, MKI-67, PDGF-A and versican, in grade 3 tumors integrin α-3, and in both groups integrin α-3 and IDH-1 mRNA expression was significantly different. Regarding protein expression, only integrin αV expression changed significantly in the prognostic groups of grade 2 tumors. Conclusions: Based on the invasion spectrum determined by this joint gene and protein expression analysis, there was a sensitivity of 87.5% and a negative predictive value of 88.9% regarding the different prognostic groups of grade 2 astrocytoma. For grade 3 tumors, the applied standard oncotherapeutic modalities apparently lacked significant anti-invasive effects.

## 1. Introduction

Astrocytic tumors are tumors of glial origin and are considered the most common histological subtype of primary central nervous system tumors. Diffuse astrocytic and oligodendroglial tumors are regarded as the second most common primary intracranial tumor type after meningioma (the incidence rate is 4.5/100,000 vs. 9.85/100,000 people), while diffuse astrocytomas are considered the third most common type among malignant central nervous system tumors (0.46/100,000 people) [1]. The nomenclature and classification of these tumors underwent significant changes over the last decade. It is important to distinguish between two types of astrocytic tumors that vary in terms of histology, molecular genetics, and prognostics: (1) circumscribed astrocytoma with relatively more indolent growth potential and (2) diffuse astrocytoma with an infiltrative character. The former type includes, among others, pilocytic astrocytoma, while the latter group includes astrocytoma of different grades (grades 2, 3, and 4) as well as glioblastoma. In the latter type, adult and pediatric subgroups can be distinguished. In terms of survival, grade 2 and grade 3 astrocytic tumors are characterized by median survival times of 5–8 years and 3 years, respectively, while glioblastoma is characterized by a very disappointing median survival time of 12–18 months [2,3,4].

For infiltrative diffuse astrocytoma, the determination of tumor grade translates to differences in prognosis as well as in oncotherapy. However, tumor grades defined on the basis of classic histological characteristics have been significantly reshaped by the inclusion of molecular genetic parameters through integrative diagnostics, as detailed in the most recent WHO classification [3]. Essential criteria for the diagnosis of astrocytic tumors include, in addition to infiltrative histopathological features, the presence of gain-of-function mutations of *IDH1* or *IDH2* and loss-of-function mutations of *ATRX* as well as the lack of 1p19q co-deletion, the latter being a diagnostic marker of oligodendroglioma. The presence of the loss-of-function *TP53* mutation is also a known characteristic. The significance of molecular markers is exemplified by the *homozygous* deletion of *CDKN2A* and/or *CDKN2B*, which, if confirmed, is clinically considered a molecular glioblastoma despite the histological features [3].

In diffusely infiltrating astrocytic tumors, the extent of surgical resection demonstrates the strongest correlation with the prognosis of patients [5,6]. However, there are significant limitations to this goal. First, it is technically impossible to completely resect the tumor due to the widely infiltrative nature of the tumor cells. Second, the extent of surgical radicality should always be balanced with the preservation of neurological functions (maximal safe resection) [7].

Infiltration is a widely studied phenomenon determined by the quantitative and qualitative differences in extracellular matrix (ECM) molecule expressions and the interactions among the ECM molecules [8,9,10,11,12,13]. The dynamic re-modeling of the tumor cells, the tumor stroma, and the ECM of the peritumoral brain tissue allows cells to migrate distances of up to several centimeters away from the primary tumor mass site [14]. This migration may most commonly occur along white matter fiber bundles (e.g., the corpus callosum), the subependymal region, and the brain vasculature [13,15,16].

The role of the ECM is mostly studied in higher-grade glial tumors, especially glioblastoma. However, considering the change in classification and the quest for a therapeutic consensus for lower-grade glial neoplasms, the study of lower-grade astrocytic tumors as well as that of the relationship between therapeutic approaches and ECM molecules provides valuable additional information [17,18,19].

Our research group has been studying the role of ECM molecules for decades, describing both their quantitative and qualitative differences and their prognostic roles in primary and secondary intracranial tumors and in the prognostic groups of glioblastoma. Our research group’s findings were used to define the ‘invasion spectrum’ in specific entities and prognostic groups [10,20]. The invasion spectrum was defined—by jointly evaluating the differences in the gene and the protein expression levels of ECM molecules—as a marker signifying invasion potential, which is characteristic of the group of interest and has been shown to have prognostic significance in glioblastoma [10].

This study sought to determine the prognostic and differential diagnostic role of the invasion spectrum in grade 2 and grade 3 astrocytomas. Additionally, the impact of oncotherapy on the invasion spectrum of grade 3 tumors was investigated.

## 2. Materials and Methods

Two subgroups of astrocytic tumors with infiltrative spreading patterns were analyzed: grade 2 and grade 3 astrocytomas. For grade 2 astrocytomas, we compared samples with different prognoses, whereas for grade 3 astrocytomas, we compared the results of samples from the first surgery with samples from patients with recurrent tumors, who had already received radiotherapy and chemotherapy. We concentrated on comparing the unique expression and invasion pattern (invasion spectra) of different ECM molecules, which play an important role in peritumoral infiltration.

For grade 2 astrocytomas, our aim was to understand the role of the invasion spectrum for prognostic purposes. For low-grade tumors, survival data was used as the basis for classification into prognostic groups: patients with an overall survival of at least 40 months were classified as having a good prognosis (N = 11), while patients who died earlier were classified into the group with a poor prognosis (N = 8). Some patients with an average survival of above 40 months were classified as having a poor prognosis due to bifrontal or multilobular involvement within the same hemisphere (No. 821, 1013, and 1042) or bifocality (No. 821—the cerebellum and brain stem).

For grade 3 glioma, seven samples from the first surgery were used as group A, while in group B, samples were removed from patients during reoperation after oncological treatments (recurrence of a residual tumor after surgery, radiotherapy, and chemotherapy).

The follow-up period was between January 2003 and February 2020, and all cases with grade 2 and grade 3 astrocytomas were treated and followed up at the Department of Oncology, University of Debrecen. All the samples processed were provided by the Brain Tumor and Tissue Bank, Department of Neurosurgery, University of Debrecen, following approval of the study by the Hungarian Scientific and Research Ethics Committee (ETT TUKEB); project identification code: 51450/2015/EKU (0411/15).

For each group, a histopathological comparison of astrocytic tumors was performed, and tumor grading was carried out by a qualified neuropathologist in keeping with the applicable protocols at the time of diagnosis. None of the cases had 1p/19q co-deletion, which is an oligodendrogioma marker.

Statistical analyses, including Mann–Whitney and Student’s *t*-test, were performed. Machine learning was used to further analyze the data, and linear discriminant analysis (LDA) was used as a statistical classifier to differentiate tumor samples using expressional data.

At the gene expression level, we assessed the invasion panel of 25 ECM molecules involved in tumor invasion. From this panel, 22 molecules and 23 molecules, respectively, were identified in the grade 2 astrocytoma group and the grade 3 astrocytoma group (Table 1).

The mRNA expression of 22 molecules [Gr. 2], and 23 molecules [Gr. 3] (Table 1) was measured in the tumor samples using quantitative reverse transcriptase real-time PCR. The flash-frozen samples were first crushed and then homogenized using TRI reagent (Invitrogen, Carlsbad, CA, USA). The whole RNA content was isolated according to the manufacturer’s instructions and NanoDrop ND-1000 spectrophotometer (NanoDrop Technologies, Wilmington, DE, USA) was used to measure the amount of mRNA. Then single-stand complement DNA was synthesized using High-Capacity cDNA Archive Kit RNasin (Applied Biosystems, Carlsbad, CA, USA). The 100 ng of synthesized cDNA was loaded onto each well of microfluidic cards to perform TaqMan Low Density Arrays (TLDA) (Applied Biosystems, 7900HT real-time PCR system with Micro Fluidic Card upgrade, Applied Biosystem, Carlsbad, CA, USA). The cycle threshold (Ct) value was determined using SDS v2.1 software (Applied Biosystem, Carlsbad, CA, USA). The glycerin-aldehyde-3-phosphate dehydrogenase housekeeping gene was used as the inner standard and reference gene to calculate delta-Ct values. 2-Ct values were used to compare mRNA expression values. Glial fibrillary acidic protein was used to confirm glial origin, Ki-67 proliferation marker was used to confirm a sufficient amount of malignant cells.

With respect to protein expression, 10 ECM molecules and 7 ECM molecules, respectively, selected after having mRNA expression results, were identified among the prognostic groups of grade 2 astrocytomas and the grade 3 group, out of which comparisons of tumor cell and extracellular matrix staining intensities were completed for 6 ECM molecules (Supplementary Materials Table S3).

Semiquantitative protein expression was measured by immunohistochemistry (IHC). The flash-frozen samples were submerged in 4% paraformaldehyde solution overnight at 4 °C, then embedded in paraffin. IHC measurements were performed in the laboratories at the Department of Anatomy, Histology, and Embryology. Table 1 contains the molecules that were labeled using IHC to confirm the findings of qRT-PCR measurements. The slides were first deparaffinated using xylene solution, then rehydrated using a series of ethanol solutions with decreasing concentrations. Heat-induced antigen retrieval was then performed in citrate buffer (pH 6.0). Primary antibodies were diluted according to the manufacturer’s instructions and then incubated over 48 h. Secondary fluorescent antibodies were diluted (1:500) and incubated overnight. A 1:2000 dilution DAPI was used for staining, then slides were sealed using Hydromount, and confocal microscopy was used to evaluate staining. Supplementary Materials Table S3 lists the primary antibodies used for IHC.

## 3. Results

### 3.1. Grade-2 Astrocytoma

#### 3.1.1. Clinico-Pathological Results

Grade-2 astrocytoma patients with poor prognosis were categorized as Group A (*n* = 8), while patients with better prognosis were classified as Group B (*n* = 11). The clinical parameters were compared between groups in terms of age, localization, side of the tumor, the extent of resection in cases of primary surgical excision, and reoperation rate. The examined clinical characteristics of the patients were not statistically different between the two groups (Table 2).

#### 3.1.2. Clinical Follow-Up—Survival Data

In terms of survival data, we compared the overall survival (OS) and the progression-free survival (PFS) of patients. Detailed follow-up data of patients was analyzed to ensure an adequate comparison (Supplementary Materials Table S1A,B).

For each patient, we determined the different PFS times observed during the course of the disease, which were then ranked chronologically. In each case, these survival times were differentiated according to radiologically and/or clinically proven progression requiring further therapeutic intervention. PFS 1 is defined as the time from diagnosis until the first progression. PFS 2 indicates the period from the first progression to the next clinically or radiologically relevant disease progression in addition to PFS 1 time, or, in other terms, the time from diagnosis until the second clinically or radiologically proven disease progression. The comparison of PFS 1 values showed no significant difference (*p* = 0.45).

**Figure 1:**
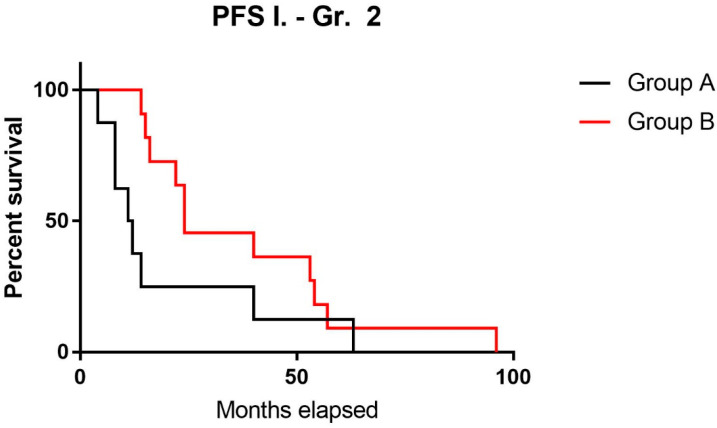
Kaplan–Meier curve in terms of progression-free survival-1 time of different prognostic groups in Grade-2 astrocytomas. Group A: patients with poor prognosis; Group B: patients with better prognoses.

It should be noted that for three cases in the group with a better prognosis, repeat operations were performed mainly for reasons affecting quality of life (epileptic seizures) and not due to significant tumor progression. In all three cases, the repeat operation was performed within four months of the first surgery, which can, thus, be considered a bias factor for PFS. Accordingly, for these patients, our calculations used the PFS 1 + PFS 2 times shown in Supplementary Materials Table S1A,B as the time to primary progression. By eliminating this bias, a significant difference (*p* = 0.02 *) in PFS was demonstrated between the two groups (Figure 1). A comparison of PFS 2 times was possible for seven cases in each group, and a significant difference (*p* = 0.04 *) between the two groups was demonstrated also for this parameter.

#### 3.1.3. mRNA Expression

We performed a unique comparison between prognostic groups in terms of the mRNA expression of each of the 22 ECM molecules involved in invasion.

According to the data detailed in Table 3, a significant difference in gene expression could be verified for six molecules: HMMR/CD168 (*p* = 0.02 *), IDH-1 (*p* = 0.009 **), Laminin α-5 (*p =* 0.03 *), MKI-67 (*p* = 0.03 *), PDGFA (*p* = 0.04 *), and versican (*p* = 0.03 *).

#### 3.1.4. Protein Expression

For the 10 ECM molecules that were part of our invasion panel, we performed an immunohistochemical study to assess their protein expression values. Based on the biological functions of these molecules, in the case of four molecules, only the ECM was evaluated, while for the other six molecules, both the ECM and the tumor cells were evaluated using immunohistochemistry (Table 4) [21]. A significant difference between the two prognostic groups could be verified by the labeling intensity of integrin αV tumor cells (*p* = 0.04 *). The concordant changes in protein expression results with the values detected at the gene expression can be observed in all the evaluated parameters of integrin αV, brevican, CSPG-5, versican, integrin β-5, and CD44 and only in tumor staining in the case of MDM2, MMP2, and FLT-4 (Table 4).

#### 3.1.5. Invasion Spectrum Analysis

Linear discriminant analysis was performed to evaluate the expression pattern of all ECM molecules in a given prognostic group using each individually assessed ECM molecule expression to differentiate the unique role of a given ECM molecule in the determination of prognostic groups.

**Figure 2:**
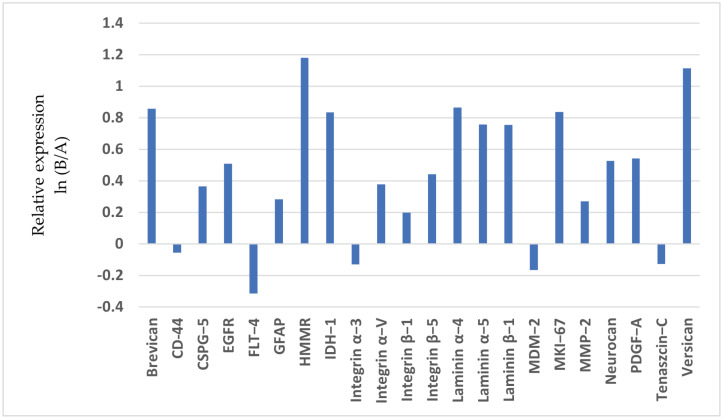
Invasion spectrum of invasion-related extracellular matrix molecules in case of Grade-2 diffuse astrocytomas. Relative expression: Quotient of average mRNA expression of each ECM molecule’s natural logarithm (ln *x*).

The molecules were ranked according to their highest contribution and, in turn, their potential to improve differentiation: integrin β-1, integrin α-V, integrin β-5, CSPG-5, HMMR/CD168, CD44, and EGFR (Table 5; Figure 2).

The evaluation of the mRNA expression of ECM molecules by linear discriminant analysis as a statistical classifier helped the determination of additional derived parameters. As a result, prognostic clustering based on this expression pattern has a sensitivity of 87.5% and a negative predictive value of 88.9% (Table 6).

### 3.2. Grade-3 Astrocytoma

#### 3.2.1. Clinico-Pathological Results

For Grade-3 astrocytomas, Group A included cases where the tumor specimen for the molecular biological analysis was taken at the time of the first tumor surgery and no prior radio-oncotherapy was performed (primary group, *n* = 12), while Group B included cases where the removal of the samples was performed at the second operation following tumor progression after radiotherapy or chemotherapy (treated group, *n* = 9) (Supplementary Materials Table S2A,B).

On evaluating the clinico-pathological data, no significant difference was found between Groups A and B in any of the examined parameters: average age (*p* = 0.1), dominant or non-dominant site of the lesion (*p* = 0.37), localization of the lesion within the lobe (*p* = 0.65), and the extent of resection at primary surgical excision (*p* = 0.49) (Table 7).

In the case of Group B, radiation therapy was applied in five cases, chemotherapy in one case, and combined radio-oncotherapy in three cases (Supplementary Materials Table S2A,B).

#### 3.2.2. Clinical Follow-Up and Survival Data

Comparing the two groups, no significant differences were detected with regard to the clinical characteristics (Table 7).

#### 3.2.3. mRNA Expression

We performed a unique comparison of the mRNA expression of 23 ECM molecules involved in invasion in samples of grade 3 astrocytoma patients (Table 8).

Based on the data presented in Table 8, a significant difference (*p* = 0.04 *) in gene expression between the primary tumor and the recurrent tumor group was observed for the integrin α-3 molecule. Although it was the only molecule with the required level of statistical significance, there were differences in the order of magnitude in the mean expression values, which are shown as the ratio of these values in the fold change column. These results clearly show that the mean gene expressions of MMP-9, EGFR, CD44, and MDM-2 also differ tremendously between the two patient groups.

**Figure 3:**
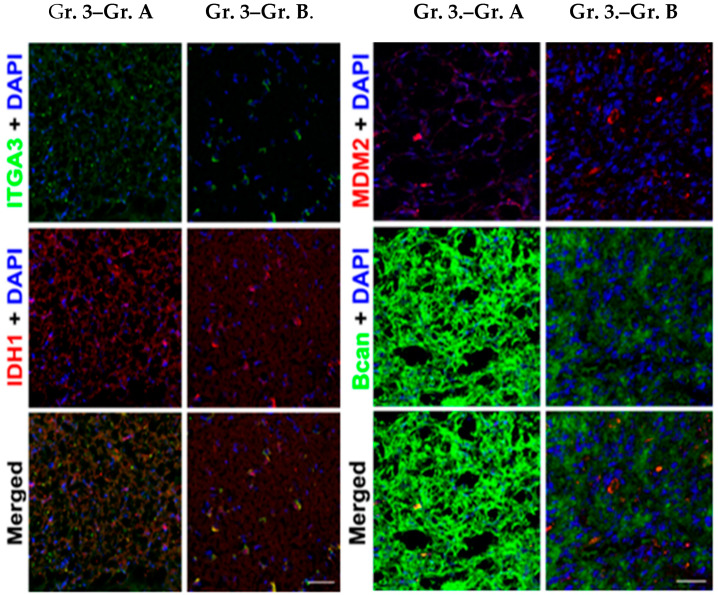
Immunohistochemical staining of integrin α-3 and IDH-1 (**left**) and brevican and MDM2 (**right**) in Gr 3 astrocytaer tumors. The patient in grade 3 group A [No. 1521-Supplementary Materials Table S2A] did not receive any previous treatment, while the patient in grade 3 group B [No. 501-Supplementary Materials Table S2B] received whole brain radiation therapy (WBRT).

In the case of grade 3 astrocytomas, no IHC staining amenable to statistical analysis could be performed, and thus it is solely qualitative for illustrative purposes (Figure 3).

#### 3.2.4. Invasion Spectrum Analysis

According to the statistical classification by the linear discriminant analysis, the molecules GFAP, HMMR, CD44, integrin α-3, IDH-1, and integrin α-V had the highest differentiation potential in this order. The sensitivity of group identification by linear discriminant analysis was 85.7%, while the negative predictive value was 88.9% (Table 6).

### 3.3. Comparison of Different Grades (Grade 2 and Grade 3)

#### 3.3.1. Clinico-Pathological Results

As part of the comparative study of tumor grades, the group of patients with poor prognosis with grade 2 tumors and the group of patients with surgery for the primary grade 3 tumors were compared; moreover, additional information was provided for these two clinically difficult-to-distinguish groups of patients. In terms of clinico-pathological features, a significant difference could only be shown in the average age of the patients (*p* = 0.003 **) (Table 9).

#### 3.3.2. mRNA Expression

In the comparison of mRNA expressions, significant differences were verified for CSPG-5 (*p* = 0.02 *), IDH-1 (*p* = 0.01 *), and integrin α-3 (*p* = 0.0003 ***) (Table 10).

#### 3.3.3. Invasion Spectrum Analysis

The MDM2, HMMR, integrin-β5, and brevican molecules represented the greatest potential for differentiation, in that order, according to the statistical classification performed by the linear discriminant analysis (Table 10).

The separation of astrocytoma of different grades using the invasion spectrum can be considered highly accurate, with a sensitivity of 93.7% and a specificity of 100% (Table 6).

## 4. Discussion

Our study groups were defined to provide answers to three major issues of practical importance.

### 4.1. Prognostic Analysis of Grade 2 Tumors

For low-grade astrocytomas, the definition of risk groups may influence postoperative oncological care. Currently, there is no clear scientific position on whether low-grade glial tumors require immediate postoperative radiotherapy and/or oncotherapy and, if so, for which group of patients should this mode of treatment be the first choice. Moreover, there is no clarity on whether a watchful waiting strategy until progression is sufficient in other respects [22,23,24]. In this study comparing the prognostic groups of grade 2 astrocytoma, the sensitivity of our prognostic grouping based on expression pattern is 87.5%, while the negative predictive value is 88.9% based on a linear discriminant analysis (Table 6). In conclusion, the invasion spectrum proved to be a suitable tool for the selection of grade 2 astrocytoma in the group with poor prognosis, and its routine use may make the differentiation of these risk groups feasible.

### 4.2. Analysis of Grade 3 Tumors in Terms of the Impact of Oncotherapy on the Invasion Spectrum

With regard to the nature of the treatment strategy, the number of therapeutic target mechanisms and target molecules, as well as the impact of treatment on patients’ overall survival and quality of life, there has been no significant progress in oncotherapy for glial tumors in recent years. Complicating factors for devising an appropriate treatment strategy are, in the case of radiotherapy, the definition of an appropriate target volume (limited radio-morphological features) and quality of life, in particular the preservation of long-term cognitive functions, while they include central nervous system penetration, intense tumor growth, and the selection of glioma cell clusters by therapy-resistant mutations in the case of oncotherapy [25].

Systemic oncotherapy agents temozolomide, carmustine, and the current standard of care combination therapy with procarbazine, lomustine, and vincristine (referred to as the PCV regimen) belong to the family of alkylating chemotherapeutics, but they do not seem to affect peritumoral infiltration—the main factor underlying recurrence [19,23,26]. Despite numerous clinical trials, for adult patients with glial tumors having an infiltrative spreading pattern, no anti-invasive agent is currently available in oncological treatment protocols. Based on our results, for grade 3 astrocytoma, after primary surgery and tumor recurrence after oncotherapy, it is clear that the differentiation between the two groups based on LDA values is effective, but a full evaluation of the results shows that integrin α-3 is the only molecule that shows significant differences and that the fold change values of change in expression do not indicate considerable differences in expression. Hence, our results support the hypothesis that radiotherapy and anti-proliferative chemotherapy have no significant effect on the invasion potential of glioma [27].

### 4.3. Comparison of Grade 2 and Grade 3 Tumors

In recent years, the nomenclature and diagnostic criteria for infiltrative diffuse glial processes have undergone considerable changes, as reflected by the cIMPACT-NOW working group publications and the latest WHO classification [2,3,28,29,30]. Currently, the increasingly used designation of lower-grade gliomas is considered a new concept in neuro-oncology. The creation of this concept was intended to bring grade 2 and grade 3 tumors together on a single platform, otherwise classified separately, which are, although histomorphologically distinct, considered to be related entities in terms of biological and clinical behavior. Therefore, their treatment strategy should be defined together [31]. The groups in our study, which aimed to compare tumors of different grades, were defined according to this principle. While the clear homogeneity of the two groups cannot be confirmed on the basis of the invasion spectrum, it should be emphasized that, in the comparison of individual molecules, only members of the integrin molecule family (molecules with marked invasive potential) and Ki-67 (an indicator of mitotic activity) reached the level of individual significance and that no significant individual difference was demonstrated in any other respect.

Research data regarding individual ECM molecules and the mechanism of invasiveness is most commonly available for higher-grade tumors, especially glioblastoma [32,33]. Our results were aimed at expanding the amount of information available on individual characteristic molecules in lower-grade glioma, indicating their potential use as therapeutic targets.

The role of the integrin family of molecules in peritumoral invasion has long been a focus of scientific interest. Integrins are heterodimeric cell surface transmembrane receptors of the glycoprotein family, composed of alpha and beta subunits, which also determine the specificity of the receptor toward the ligand. Grouped by ligand, they can be differentiated as collagen, laminin, RGD (arginine-glycine-aspartate) amino acid sequence-mediated dimer integrins, and leukocyte receptors. While they have no intrinsic activity, they can activate downstream signaling pathways via focal adhesion kinases (FAK) [34]. The interaction between tumor cells and the microenvironment, the stromal compartment of the extracellular matrix, is part of the invasion. It is facilitated by integrin molecules in multiple ways. These molecules also mediate angiogenic and proliferative signals by activating signaling pathways and serve as anchoring structures, among others [35].

The beta-1 subunit is considered one of the most important of the eight beta subunits, with several alpha subunits capable of forming a heterodimer in the perivascular space. In vitro experiments with a neutralizing antibody against this subunit have shown that it alone can induce a reduction in invasion potential; however, it can also potentiate the effect of neovascularization inhibitor treatment (bevacizumab) used in clinical practice [36,37,38,39]. Our study clearly confirmed the prognostic role of this molecule, and based on our results, this molecule primarily helped in the identification of the poorer prognosis group.

Integrin-αV is an integrin subunit capable of dimerization by recognizing the RGD sequence. Its ligands include fibronectin, fibrinogen, and tenascin. The role of the alpha-V subunit has been detailed mainly in high-grade glial processes [40]. Its role is illustrated by the fact that a specific neutralizing antibody against αvβ3 and αvβ5 subunits, called cilengitide, has been the subject of several studies. Based on results obtained to date, cilengitide has not been introduced to daily practice, but there is still considerable scientific interest in the neutralization of this subunit [41,42,43,44]. In our study, the role of the alpha-V subunit was a significant contributing factor in the differentiation among all three of the study groups. Our results confirmed the significant role of integrin-αV in influencing peritumoral infiltration in lower-grade glioma, and it is a promising prognostic as well as an anti-invasive molecular target for this less-studied patient group [45].

The role of integrin α-3 in invasion is confirmed by the identification of increased expression of integrin α-3 in glioma stem-like cells. A study of glioblastoma cell lines revealed that integrin α-3-positive cells showed increased expression in the areas considered stem cell niches surrounding blood vessels and were overrepresented in infiltrating cells. Moreover, integrin α-3 expression correlated with the invasion potential of these cells [46]. In tumors of glial origin, hyaluronic acid, which is a part of the group of glycosaminoglycans, plays a major role in the invasion process, correlating with a higher concentration of hyaluronic acid compared to the normal brain ECM, and its expression has been shown to be proportional to the grade [47]. Hyaluronic acid can bind HMMR (CD 168) as well as CD 44 molecules. The vast majority of available studies have also been performed in higher-grade glial processes; however, studies performed by evaluating databases including a significant number of cases independent of grade (the Cancer Genome Atlas, TCGA) have confirmed the prognostic role of CD 168 and CD 44 [48,49,50]. Our own results confirmed the prominent role of these molecules in the infiltrative processes related to lower-grade glial processes. The MDM2, HMMR, integrin-β5, and brevican molecules represented the greatest potential for differentiation; thus, the separation of astrocytoma of different grades using the invasion spectrum can be considered highly accurate, with a sensitivity of 93.7% and a specificity of 100%, based on recent investigations.

## 5. Conclusions

Determining the prognostic role of the invasion spectrum may facilitate decision-making in uncertain cases of grade 2 astrocytoma to establish an indication for postoperative oncotherapy. Furthermore, it has been confirmed that current oncotherapy does not significantly affect tumor invasion in grade 3 astrocytoma; hence, the exploration of therapeutic options in this direction is recommended. In view of the significance of molecular therapeutic targets in recent clinical trials, our study also draws attention to new target molecules that may be potentially applicable to anti-invasive therapy.

## Figures and Tables

**Table 1:** List of analyzed extracellular matrix molecules and molecular methods in the respective grades. A 2: astrocytoma grade 2, A 3: astrocytoma grade 3, +: Analyzed in the respective group; -: Not analyzed in the respective group.

No.	Invasion Panel	A 2-qRT-PCR	A 2-IF	A 3-qRT-PCR	A 3-IF
1.	Brevican	+	+	+	+
2.	CD 44	+	+	+	+
3.	CSGPG-5	+	+	+	-
4.	EGFR	+	-	+	-
5.	GFAP	+	-	+	-
6.	HMMR/CD168	+	+	+	-
7.	IDH-1	+	-	+	+
8.	Integrin α-3	+	-	+	+
9.	Integrin α-V	+	+	+	-
10.	Integrin β-1	+	-	+	-
11.	Integrin β-5	+	+	+	-
12.	Laminin α-4	+	-	+	-
13.	Laminin β-1	+	-	+	-
14.	MDM-2	+	+	+	+
15.	MKI-67	+	-	+	-
16.	MMP-2	+	+	+	-
17.	Neurocan	+	-	+	+
18.	PDGF-A	+	-	+	-
19.	Tenascin-C	+	-	+	-
20.	Versican	+	+	+	+
21.	FLT-4	+	+	-	-
22.	Laminin α-5	+	-	-	-
23.	HAS-2	-	-	+	-
24.	MMP-9	-	-	+	-
25.	Integrin α-5	-	-	+	-

**Table 2:** Clinico-pathological parameters of Gade-2 astrocytoma patients with different prognoses. PFS 1 = progression-free survival; PFS 1-mod. = progression-free survival without outliers; PFS 2 = progression-free survival until second clinically or radiologically relevant disease progression; OS = overall survival; Group A: patients with poor prognosis; Group B: patients with better prognoses; *: *p*-value is less than 0.05.

Groups	Age (Average ± SD; Years)	Localization	Side	Extent of 1st Surgical Intervention	Reoperation Rate	PFS 1 (Average ± SD; Months)	PFS 1-Mod. (Average ± SD; Months)	PFS 2 (Average ± SD; Months)	OS (Average ± SD; Months)
Gr 2-“Group A” *n* = 8	32.1 ± 6.96	Frontal: 3 Temporal: 1 Parietal: 0 Multilobular: 3 Other: 1	Right-sided: 7 Left-sided: 1	Macroscopically total: 6 Partial: 2	7/8	20.0 ± 20.61	20.0 ± 20.61	36.3 ± 34.78	54.6 ± 44.98
Gr 2-“Group B” *n* = 11	35.4 ± 10.57	Frontal: 2 Temporal: 5 Parietal: 3 Multilobular: 1 Other: 0	Right-sided: 5 Left-sided: 6	Macroscopically total: 4 Partial: 7	8/11	32.3 ± 29.34	37.7 ± 25.39	70.9 ± 37.97	85.5 ± 39.24
*p*-value	0.67	0.10	0.06	0.11	0.45	0.45	0.02 *	0.04 *	0.18

**Table 3:** mRNA expressional data of ECM molecules in prognostic groups of Grade-2 astrocytoma samples. Group A: patients with poor prognosis; Group B: patients with better prognosis. (*: *p*-value is less than 0.05, **: *p*-value is less than 0.01).

ECM Molecules	Mean ± SD mRNA Expression in Group A	Mean ± SD mRNA Expression in Group B	Greater mRNA Expression in Group	Fold Change	LDA-Value	Contribution to Decision Tree in the Direction of Group	*p*-Value
Brevican	0.37 ± 0.46	0.87 ± 0.55	B	2.35	−0.348	A	0.13
CD 44	0.13 ± 0.07	0.12 ± 0.07	A	0.92	−3.910	A	0.72
CSGPG-5	0.07 ± 0.07	0.1 ± 0.07	B	1.43	−3.995	A	0.49
EGFR	0.08 ± 0.11	0.12 ± 0.08	B	1.5	3.721	B	0.12
FLT-4	0.001 ± 0.002	0.0008 ± 0.0005	A	0.8	-	-	0.56
GFAP	8.63 ± 6.75	11.46 ± 8.72	B	1.33	−3.375	A	0.35
HMMR	0.0002 ± 0.0001	0.0006 ± 0.0006	B	3.0	3.910	B	0.02 *
IDH-1	0.003 ± 0.002	0.007 ± 0.0041	B	2.33	−1.829	A	0.009 **
Integrin α-3	0.007 ± 0.002	0.0059 ± 0.003	A	0.84	2.250	B	0.78
Integrin α-V	0.06 ± 0.03	0.09 ± 0.04	B	1.5	7.091	B	0.11
Integrin β-1	0.04 ± 0.015	0.05 ± 0.03	B	1.25	−7.396	A	>0.99
Integrin β-5	0.03 ± 0.02	0.04 ± 0.03	B	1.33	4.019	B	0.18
Laminin α-4	0.014 ± 0.011	0.032 ± 0.026	B	2.29	−1.591	A	0.051
Laminin α-5	0.010 ± 0.006	0.022 ± 0.012	B	2.2	-	-	0.03 *
Laminin β-1	0.006 ± 0.006	0.013 ± 0.012	B	2.2	2.078	B	0.11
MDM-2	0.03 ± 0.03	0.027 ± 0.012	A	0.9	−0.976	A	0.54
MKI-67	0.0021 ± 0.0032	0.0048 ± 0.0061	B	2.3	-	-	0.03 *
MMP-2	0.013 ± 0.006	0.017 ± 0.008	B	1.3	−0.104	A	0.24
Neurocan	0.065 ± 0.06	0.11 ± 0.09	B	1.7	1.935	B	0.44
PDGF-A	0.017 ± 0.016	0.03 ± 0.018	B	1.76	0.645	B	0.04 *
Tenascin-C	0.1 ± 0.061	0.09 ± 0.05	A	0.9	-	-	0.84
Versican	0.14 ± 0.18	0.42 ± 0.27	B	3.0	-	-	0.03 *

**Table 4:** Immunohistochemical staining of ECM molecules in different prognostic groups of Grade-2 astrocytoma samples. *: *p*-value is less than 0.05. Group A: patients with poor prognosis; Group B: patients with better prognoses.

No.	ECM Molecule	Protein Expression in Group A [Mean ± SD]	Protein Expression in Group B [Mean ± SD]	*p*-Value	Concordant Change with mRNA Expression (Yes/No)
1.	Brevican-ECM	5.6 ± 1.1	6.6 ± 1.0	0.08	Yes
2.	CSPG5-ECM	5.4 ± 1.1	5.5 ± 1.0	0.67	Yes
3.	Versican-ECM	6.1 ± 1.4	6.4 ± 1.4	0.62	Yes
4.	Integrin β-5-ECM	5.0 ± 1.3	5.4 ± 2.0	0.81	Yes
5.	CD-44-Tumor cells	7.3 ± 2.0	6.8 ± 1.6	0.62	Yes
	CD-44-ECM	6.7 ± 1.4	6.6 ± 1.4	0.69	Yes
6.	MDM2-Tumor cells	5.4 ± 3.0	5.2 ± 2.2	>0.99	Yes
	MDM2-ECM	4.5 ± 1.2	4.7 ± 1.2	0.89	No
7.	HMMR-Tumor cells	8.1 ± 2.1	7.6 ± 2.9	0.91	No
	HMMR-ECM	5.3 ± 1.2	5.2 ± 1.7	0.91	No
8.	Integrin αV-Tumor cells	8.0 ± 1.2	9.0 ± 1.1	0.04 *	Yes
	Integrin αV-ECM	6.6 ± 0.8	7.5 ± 0.8	0.06	Yes
9.	MMP-2-Tumor cells	6.5 ± 1.7	7.6 ± 1.4	0.14	Yes
	MMP-2-ECM	5.5 ± 1.8	5.3 ± 0.9	0.84	No
10.	FLT-4-Tumor cells	5.0 ± 0.9	4.9 ± 1.9	0.87	Yes
	FLT-4-ECM	4.5 ± 1.7	4.7 ± 1.6	0.79	No

**Table 5:** Contribution of ECM molecules to prognostic group classification. x.: Insignificant contribution to decision-making.

No.	ECM Molecules	Gr. 2 Astrocytoma LDA Values	ECM Molecules	Gr. 3 Astrocytoma LDA Values
1.	Integrin β-1	−7.396	GFAP	30.709
2.	Integrin α-V	7.091	HMMR/CD168	21.611
3.	Integrin β-5	4.019	CD 44	−13.732
4.	CSGPG-5	−3.995	Integrin α-3	10.511
5.	HMMR/CD168	3.910	IDH-1	−8.970
6.	CD 44	−3.910	Integrin α-V	−8.845
7.	EGFR	3.721	Integrin β-1	−7.480
8.	GFAP	−3.375	MDM-2	−6.136
9.	Integrin α-3	2.250	HAS-2	−4.548
10.	Laminin β-1	2.078	Brevican	2.063
11.	Neurocan	1.935	CSPG-5	2.027
12.	IDH-1	−1.829	EGFR	−1.871
13.	Laminin α-4	−1.591	Integrin β-5	1.682
14.	MDM-2	−0.976	Integrin α-5	0.704
15.	PDGF-A	0.645	MKI-67	x
16.	Brevican	−0.348	MMP-2	x
17.	MMP-2	−0.104	Neurocan	x
18.	MKI-67	x	PDGF-A	x
19.	Tenascin-C	x	Tenascin-C	x
20.	Versican	x	Versican	x
21.	FLT-4	x	FLT-4	x
22.	Laminin α-5	x	Laminin α-5	x
23.	HAS-2	x	MMP-9	x
24.	MMP-9	x	Laminin α-4	x
25.	Integrin α-5	x	Laminin β-1	x

**Table 6:** Confusion matrix of mRNA gene expression linear discriminant analysis in group identification. Gr. 2-Group A: patients with poor prognoses; Gr. 2-Group B: patients with better prognoses; Gr.-3 Group A: primary patient group without prior treatment applied (primary group); Gr.-3 Group B: patients with prior treatment applied (treated group).

Parameter	Gr. 2-Group A vs. B	Gr. 3-Group A vs. B	Gr. 2 vs. Gr. 3
Sensitivity	87.5%	85.7%	93.7%
Specificity	72.7%	88.9%	100%
Positive predictive value	70.0%	85.7%	100%
Negative predictive value	88.9%	88.9%	95%

**Table 7:** Clinico-pathological parameters of Grade-3 astrocytoma patients. OS: overall survival. Group A: primary patient group without prior treatment applied (primary group); Group B: patients with prior treatment applied (treated group).

Groups	Age (Average ± SD; Years)	Localization	Side	Extent of 1st Surgical Intervention	OS (Average ± SD; Months)
Gr 2-“Group A” *n* = 8	32.1 ± 6.96	Frontal: 3 Temporal: 1 Parietal: 0 Multilobular: 3 Other: 1	Right-sided: 7 Left-sided: 1	Macroscopically total: 6 Partial: 2	54.6 ± 44.98
Gr 2-“Group B” *n* = 11	35.4 ± 10.57	Frontal: 2 Temporal: 5 Parietal: 3 Multilobular: 1 Other: 0	Right-sided: 5 Left-sided: 6	Macroscopically total: 4 Partial: 7	85.5 ± 39.24
*p*-value	0.67	0.10	0.06	0.11	0.18

**Table 8:** mRNA expressional data of ECM molecules in different groups of Gr 3 astrocytoma samples. Group A: primary patient group without prior treatment applied (primary group); Group B: patients with prior treatment applied (treated group). *: *p*-value is less than 0.05.

ECM Molecules	mRNA Expression in Group A [Mean ± SD]	mRNA Expression in Group B [Mean ± SD]	Greater mRNA Expression in Group	Fold Change (B/A)	LDA-Value	Contribution to Decision Tree in the Direction of Group	*p*-Value
Brevican	1.0712 ± 1.0548	0.7272 ± 0.77	A	0.68	2.063	A	0.43
CD 44	0.2872 ± 0.2174	0.5433 ± 0.48	B	1.89	−13.732	B	0.27
CSGPG-5	0.4502 ± 0.3992	0.2439 ± 0.18	A	0.54	2.027	A	0.22
EGFR	1.3601 ± 2.9338	0.4111 ± 0.82	A	0.30	−1.871	B	0.79
GFAP	16.0084 ± 8.0520	27.4170 ± 22.38	B	1.71	30.709	A	0.56
FLT-4	-	-	-	-	-	-	-
HAS-2	0.0027 ± 0.0012	0.0030 ± 0.003	B	1.11	−4.548	B	0.71
HMMR	0.0005 ± 0.00048	0.0004 ± 0.0004	A	0.80	21.611	A	0.37
IDH-1	0.0200 ± 0.016	0.0202 ± 0.011	B	1.01	−8.970	B	0.71
Integrin α-3	0.0341 ± 0.03	0.0134 ± 0.0057	A	0.39	10.511	A	0.04 *
Integrin α-5	0.051 ± 0.034	0.027 ± 0.031	A	0.53	0.704	A	0.15
Integrin α-V	0.0829 ± 0.032	0.1029 ± 0.05	B	1.24	−8.845	B	0.49
Integrin β-1	0.0318 ± 0.012	0.0341 ± 0.018	B	1.07	−7.480	B	0.87
Integrin β-5	0.0275 ± 0.019	0.0527 ± 0.04	B	1.92	1.682	A	0.22
Laminin α-4	0.0086 ± 0.0049	0.0077 ± 0.005	A	0.90	-	-	0.71
Laminin α-5	-	-	-	-	-	-	-
Laminin β-1	0.0048 ± 0.0025	0.0061 ± 0.004	B	1.27	-	-	0.79
MDM-2	0.0102 ± 0.0028	0.0278 ± 0.05	B	2.73	−6.136	B	0.27
MKI-67	0.0050 ± 0.003	0.0053 ± 0.006	B	1.06	-	-	0.49
MMP-2	0.0177 ± 0.014	0.0177 ± 0.018	B	1.0	-	-	0.79
MMP-9	0.0337 ± 0.06	0.0040 ± 0.004	A	0.12	-	-	0.37
Neurocan	0.1707 ± 0.15	0.2033 ± 0.1	B	1.19	-	-	0.43
PDGF-A	0.0352 ± 0.038	0.0274 ± 0.017	A	0.78	-	-	0.96
Tenascin-C	0.1011 ± 0.078	0.1127 ± 0.15	B	1.11	-	-	0.56
Versican	0.2631 ± 0.24	0.2045 ± 0.16	A	0.78	-	-	0.71

**Table 9:** Clinico-pathological parameters of Grade-2 poor prognostic patient group (Gr. 2-Group A) and Grade-3 primary patient group without prior treatment applied (Gr. 3-Group A); OS: overall survival.

Groups	Age (Average ± SD; Years)	Localization	Side	Extent of 1st Surgical Intervention	OS (Average ± SD; Months)
Gr 2-“Group A” *n* = 7	54.4 ± 7.44	Frontal: 2 Temporal: 2 Parietal: 1 Multilobular: 2 Other: -	Right-sided: 5 Left-sided: 2	Macroscopically total: 4 Partial/biopsy: 3 Not specified: 0	48.9 ± 70.66
Gr 3-‘Group A” *n* = 9	43.0 ± 13.09	Frontal: 2 Temporal: 3 Parietal: 0 Multilobular: 4 Other: -	Right-sided: 8 Left-sided: 1	Macroscopically total: 3 Partial/biopsy: 5 Not specified: 1	45.8 ± 34.78
*p*-value	0.1	0.65	0.37	0.49	0.46

**Table 10:** mRNA expressional data of ECM molecules in the Grade-2 poor prognostic patient group (Gr. 2-Group A) and the Grade-3 primary patient group without prior treatment applied (Gr. 3-Group A). (*: *p*-value is less than 0.05, ***: *p*-value is less than 0.001).

ECM Molecules	mRNA Expression in Gr 2-Group A [Mean ± SD]	mRNA Expression in Gr 3-Group A [Mean ± SD]	Greater mRNA Expression in Group	Fold Change (Gr. 3/Gr. 2)	LDA-Value	Contribution to Decision Tree in the Direction of Group	*p*-Value
Brevican	0.37 ± 0.46	1.0712 ± 1.0548	Gr. 3	2.90	6.81	Gr. 3	0.19
CD 44	0.13 ± 0.07	0.2872 ± 0.2174	Gr. 3	2.21	−2.54	Gr. 2	0.34
CSGPG-5	0.07 ± 0.07	0.4502 ± 0.3992	Gr. 3	6.43	1.44	Gr. 3	0.02 *
EGFR	0.08 ± 0.11	1.3601 ± 2.9338	Gr. 3	17.0	2.7	Gr. 3	0.34
GFAP	8.63 ± 6.75	16.0084 ± 8.0520	Gr. 3	1.85	1.11	Gr. 3	0.054
FLT-4	-	-	-	-	-	-	-
HAS-2	-	-	-	-	-	-	-
HMMR	0.0002 ± 0.0001	0.0005 ± 0.00048	Gr. 3	2.5	−7.39	Gr. 2	0.19
IDH-1	0.003 ± 0.002	0.0200 ± 0.016	Gr. 3	6.67	2.6	Gr. 3	0.01 *
Integrin α-3	0.007 ± 0.002	0.0341 ± 0.03	Gr. 3	4.87	3.44	Gr. 3	0.0003 ***
Integrin α-5	-	-	-	-	-	-	-
Integrin α-V	0.06 ± 0.03	0.0829 ± 0.032	Gr. 3	1.38	1.47	Gr. 3	0.23
Integrin β-1	0.04 ± 0.015	0.0318 ± 0.012	Gr. 2	0.8	5.02	Gr. 3	0.34
Integrin β-5	0.03 ± 0.02	0.0275 ± 0.019	Gr. 2	0.92	−7.27	Gr. 2	0.87
Laminin α-4	0.014 ± 0.011	0.0086 ± 0.0049	Gr. 2	0.61	7.2	Gr. 3	0.34
Laminin α-5	-	-	-	-	-	-	-
Laminin β-1	0.006 ± 0.006	0.0048 ± 0.0025	Gr. 2	0.8	−0.6	Gr. 2	0.78
MDM-2	0.03 ± 0.03	0.0102 ± 0.0028	Gr. 2	0.34	−7.61	Gr. 2	0.12
MKI-67	0.0021 ± 0.0032	0.0050 ± 0.003	Gr. 3	2.38	6.4	Gr. 3	0.02 *
MMP-2	0.013 ± 0.006	0.0177 ± 0.014	Gr. 3	1.36	−5.8	Gr. 2	0.46
MMP-9	-	-	-	-	-	-	-
Neurocan	0.065 ± 0.06	0.1707 ± 0.15	Gr. 3	2.63	-	-	0.19
PDGF-A	0.017 ± 0.016	0.0352 ± 0.038	Gr. 3	2.07	-	-	0.28
Tenascin-C	0.1 ± 0.061	0.1011 ± 0.078	Gr. 3	1.01	-	-	0.99
Versican	0.14 ± 0.18	0.2631 ± 0.24	Gr. 3	1.88	-	-	0.19

## Data Availability

The data presented in this study are available on request from the corresponding author due to ethical reasons.

## Supplementary Materials

The following supporting information can be downloaded at: https://www.mdpi.com/article/10.3390/brainsci14111157/s1, Table S1. (A) Patients with Grade 2 astrocytoma in poor prognostic group, (B) Patients with Grade 2 astrocytoma in better prognostic group, Table S2. (A) Previously untreated patients with Grade 3 astrocytoma, (B) Previously treated patients with Grade 3 astrocytoma, Table S3: List of primary antibodies used for immunohistochemical staining.
