# Supplementary material for: Prognostic Role of Invasion-Related Extracellular Matrix Molecules in Diffusely Infiltrating Grade 2 and 3 Astrocytomas
Source: Brain Sci. 2024 Nov 20;14(11):1157. doi: 10.3390/brainsci14111157 (PMC11592374; doi:10.3390/brainsci14111157)
Supplement: Supplementary file 1 [file brainsci-14-01157-s001.zip › brainsci-3293829-supplementary-done.pdf]

brain sciences

MDPI

Supplementary Material

Table S1A. Patients with Grade 2 astrocytoma in poor prognostic group.

No.: patient identification number; OS: overall survival; PFS1-7: progressio-free survival, time periods of disease progression in term of clinical or radiological evidence; P: macroscopically partial tumor excision; T: macroscopically total surgical excision; FBRT: focal brain radiation therapy; PCV: chemotherapy regimen [procarbazine, lomustine, vincristine], TMZ: temozolomide chemotherapy; BCNU: carmustine chemotherapy; BSC: best supportive care, Tx = applied treatment.

No.	OS (Months)	Age (Years)	Side and Localization	1st Tx	PFS1/Tx (Months)	PFS2/Tx (Months)	PFS3/Tx (Months)	PFS4/Tx (Months)	PFS5/Tx (Months)	PFS6/Tx (Months)	PFS7/Tx (Months)	MIB-1 LI
1013	129	23	Bifrontal	Surgical resection (T)	63/Surgical resection (P)	42/FBRT	16/TMZ	8/BSC	-	-	-	4–6
1042	96	39	Left/Multilob.	Surgical resection (T)	8/Surgical resection (P)	21/FBRT	23/Gamma-knife surgery + TMZ	13/Re-irradiation (FBRT)	5/PCV	20/Surgical resection (P − Histology: GBM) + Avastin	4/BSC	<1%
1081	76	33	Right/Frontal—Corpus callosum inf.	Surgical resection (T)	14/FBRT	6/TMZ	26/Gamma-knife surgery	2/PCV	1/Surgical excision (P − Histology: Radiation necrosis) + PCV	1/PCV	23/BSC	3–5%
1196	8	34	Right/Frontal	Surgical resection (P)	4/PCV	-	-	-	-	-	-	<2%
1216	12	25	Right/Frontal	Surgical resection (T)	11/surgical resection (T – Histology: GBM) + Stupp - protocoll	ND (KPS: 100)	-	-	-	-	-	4%
1292	12	37	Right/Thalamus	Biopsy + ETV	8/surgical resection (P) + WBRT	(3/VP – shunt)	-	-	-	-	-	1–2%
821	71	25	Bifrontal—Infatentorial	Surgical resection (T)	40/surgical resection (P) + WBRT	20/BCNU	3/TMZ	ND (KPS:40)	-	-	-	<4%
902	34	41	Right/Temporal	Surgical resection (T)	12/surgical resection (P) + WBRT	8/BCNU	9/TMZ	2/BSC	-	-	-	3–8%

Table S1B. Patients with Grade 2 astrocytoma in good prognostic group.

No.: patient identification number; OS: overall survival; PFS1-5: progression-free survival, time periods of disease progression in term of clinical or radiological evidence; P: macroscopically partial tumor excision; T: macroscopically total surgical excision; FBRT: focal brain radiation therapy; PCV: chemotherapy regimen [procarbazine, lomustine, vincristine], TMZ: temozolomide chemotherapy; BCNU: carmustine chemotherapy; BSC: best supportive care, Tx = applied treatment

No.	OS (Months)	Age (Years)	Side and Localization	"Wait and See" Before the 1st Surgical Excision	1st Tx	PFS1/Tx (Months)	PFS2/Tx (Months)	PFS3/Tx (Months)	PFS4/Tx (Months)	PFS5/Tx (Months)	MIB-1 LI
802	74	53	Right/Temporal	YES - 14 months	Surgical excision (P)	14 months – wait and see/surgical excision (P)	32/Surgical excision (P) + FBRT	27/BSC		-	<2%
858	114	23	Right/Frontal	NO	Surgical excision (T)	24/Surgical excision (T) + FBRT	82/TMZ	4 hónap/BCNU	-	-	NS
1023	146	55	Left/Temporal	NO	Surgical excision (P) + PCV	57/Surgical excision (P)#	-	-	-	-	NS
1071	83	34	Left/Multilob.	NO	Surgical excision (P)	22/Surgical excision (T) + WBRT	50/TMZ	-	-	-	<2%
1096	96	37	Right/Temporal	NO	Surgical excision (P)	4/Surgical excision (P) – cause: epilepsy	36/Surgical excision (P)	12/Surgical excision (P) + WBRT (not accepted)	15/VP shunt	12/BSC	<2%
1156	68	30	Left/Frontal	NO	Surgical excision (P)	4/FBRT – cause: epilepsy	12/TMZ	23/TMZ re-induction	-	-	3%
1211	158	26	Left/Parietal	YES—96 months	Surgical excision (P)	96 – wait and see/Surgical excision (P)	41/Surgical excision (P – Hist.: Gr. III) + FBRT + PCV	3/TMZ	-	-	1–6%
1253	54	30	Left/Parietal	NO	Surgical excision (T)#	-	-	-	-	-	<1%
1260	54	31	Left/Temporal	NO	Surgical excision (T)	3/Surgical excision (T) – cause: epilepsy	12/Surgical excision (P)	-	-	-	1%
1295	53	28	Right/Parietal	NO	Surgical excision (T)	53/FBRT*	-	-	-	-	1–2%
1349	40	42	Right/Temporal	NO	Surgical excision (P)	24/Surgical excision (P – Histology: Gr. III astrocytoma)	15/Surgical excision (P) + FBRT + TMZ*	-	-	-	NS

Table S2A. Previously untreated patients with Grade 3 astrocytoma.

No.: patient identification number; OS: overall survival; PFS1-4: progression-free survival, time periods of disease progression in term of clinical or radiological evidence; P: macroscopically partial tumor excision; T: macroscopically total surgical excision; FBRT: focal brain radiation therapy; PCV: chemotherapy regimen [procarbazine, lomustine, vincristine], TMZ: temozolomide chemotherapy; BCNU: carmustine chemotherapy; BSC: best supportive care; ND: No further follow-up data is available; NS: not specified, Tx = applied treatment. Actual tissue specimen: **Bold.**

No.	OS (Months)	Age (Years)	Side and Localization	1st Surgical Tx (Extent)	Postoperative Tx	PFS1/Tx (Months)	PFS2/Tx (Months)	PFS3/Tx (Months)	PFS4/Tx (Months)	Reoperation During Follow-Up	Tissue Specimen from Operation No.	Applied Tx Before	MIB-1 LI
282.	27	47	Right/Multilob.	**Macroscopically total**	WBRT	10/Surgical resection (T) + BCNU	13/BSC	-	-	Yes	1.	No	2–8%
325.	12	56	Right/Parietal	**Macroscopically total**	WBRT	6/Surgical resection (T – Hist.: GBM) + TMZ	4/re-irradiation	ND	-	Yes	1.	No	65–80%
704.	51	54	Left/Temporal	Biopsy (Hist.: Gr. II glioma susp.)	-	7/**Surgical resection (P)** + WBRT	5/TMZ	6/Surgical resection (P)	13/BSC	Yes	2.	No	10–25%
1386.	21	54	Right/Frontal	**Macroscopically partial**	FBRT	13/Surgical resection (T) + TMZ	7/BSC	-	-	Yes	1.	No	1–10%
1515.	11	44	Left/Temporal	**Macroscopically partial**	WBRT	-	-	-	-	No	1.	No	1–5%
1521.	14	66	Right/Multilob.	**Macroscopically total**	FBRT	4/TMZ	4/BSC			No	1.	No	8–10%
1542.	206	60	Right/Frontal	Macroscopically total (Hist.: Gr II–Gr. III mixed)	-	192 months/**Surgical resection (T)** + postponded WBRT	ND	-	-	Yes	2.	No	5–6%

Table S2B. Previously treated patients with Grade 3 astrocytoma.

No.: patient identification number; OS: overall survival; PFS1-4: progression-free survival, time periods of disease progression in term of clinical or radiological evidence; P: macroscopically partial tumor excision; T: macroscopically total surgical excision; FBRT: focal brain radiation therapy; PCV: chemotherapy regimen [procarbazine, lomustine, vincristine], TMZ: temozolomide chemotherapy; BCNU: carmustine chemotherapy; BSC: best supportive care; Tx = applied treatment, ND: No further follow-up data is available; NS: not specified; Applied Tx before investigation: *Italic Underlined;* Actual tissue specimen: **Bold,.**

**No.**	**OS (Months)**	**Age (Years)**	**Side and Localization**	**1st Surgical Tx (Extent)**	**Postoperative Tx**	**PFS1/Tx (months)**	**PFS2/Tx (Months)**	**PFS3/Tx (Months)**	**PFS4/Tx (Months)**	**Tissue Specimen from Operation No.**	**Applied Tx Before**	**MIB-1 LI**
**503**	27	47	Right/Multilob.	Macroscopically total	*WBRT*	10/**Surgical resection (T)** + BCNU	13/BSC	-	-	2.	Yes	2-8 %
**781**	19	61	Right/Temporal	Macroscopically total (Hist.: GBM susp.)	*Stupp-protocoll*	6/**Surgical resection (T – Hist.: AIII)** + BCNU	9/Surgical resection (T – Hist: GBM)	4/Surgical resection (T – Hist: GBM)	1/BSC	2.	Yes	30-55 %
**820**	17	31	Right/Temporal	Macroscopically partial	*FBRT*	15/**Surgical resection (P)** + TMZ	ND	-	-	2.	Yes	NS
**837**	73	53	Left/Multilob.	Macroscopically partial	*PCV*	63/**Surgical resection (P)** + FBRT	6/TMZ	ND		2.	Yes	NS
**1067**	50	25	Right/Multilob.	Macroscopically partial	*WBRT + CCNU*	15/**Surgical resection (P)** + TMZ	9/Stereotax. gamma-irradiation	24/Surgical resection (P) + TMZ-reinduction	ND	2.	Yes	<5 %
**1255**	13	48	Right/Multilob.	Macroscopically total	*FBRT*	7/**Surgical resection (T)** + TMZ	5/BSC	-	-	2.	Yes	5-8 %
**1321**	84	26	Right/Frontal	Macroscopically partial (Hist: Gr. II)	*Irradiation*	84/**Surgical resection (P)** + TMZ	ND	-	-	2.	Yes	5%
**1420**	108	41	Right/Temporal	NS (Hist.: Gr. II)	-	60/Surgical resection (NS – Hist.: Gr. III) + *irradiation*	24/*TMZ*	24/**Surgical resection (T)**	ND	3.	Yes	1-70 %
**1475.**	21	55	Right/Frontal	Macroscopically partial	*FBRT*	12/**Surgical resection (T)** + TMZ	7/BSC	-	-	2.	Yes	10%

Table S3. List of primary antibodies used for immunohistochemical staining.

No.	Name	Manufacturer	Antibody Code	Dilution	Clonality	Host	Positive Control
1.	Brevican	Novus Biologicals (Littleton, CO, USA)	NBP1-89992PEP	1:200	Polyclonal	Rabbit	Rat brain
2.	CD 44	Abcam (Cambridge, UK)	AB16728	1:500	Monoclonal	Mouse	Human tonsil
3.	CSPG-5 (neuroglycan C)	Biorbyt (Cambridge, UK)	ORB157961	1:250	Polyclonal	Rabbit	Rat brain
4.	FLT-4/VEGFR-3	Santa Cruz Biotechnology (Dallas, TX, USA)	SC-514825	1:250	Polyclonal	Rabbit	Human kidney
5.	HMMR (CD168, RHAMM)	Abcam (Cambridge, UK)	AB110075	1:200	Polyclonal	Rabbit	Human tonsil
6.	Integrin alpha V chain	Bioss Antibodies (Woburn, MS, USA)	BS-2203R	1:250	Polyclonal	Rabbit	Human kidney
7.	Integrin beta 5 chain	Novus Biologicals (Littleton, CO, USA)	NBP1-88117	1:250	Polyclonal	Rabbit	Human tonsil
8.	MDM2	Abcam (Cambridge, UK)	AB16895	1:400	Monoclonal	Mouse	Rat brain
9.	MMP-2	Origene Technologies (Rockville, MD, USA)	TA806846	1:200	Monoclonal	Mouse	Human tonsil
10.	Versican	Abcam (Cambridge, UK)	AB177480	1:150	Monoclonal	Rabbit	Rat brain

Disclaimer/Publisher's Note: The statements, opinions and data contained in all publications are solely those of the individual author(s) and contributor(s) and not of MDPI and/or the editor(s). MDPI and/or the editor(s) disclaim responsibility for any injury to people or property resulting from any ideas, methods, instructions or products referred to in the content.
